# Neural matrix factorization++ based recommendation system

**DOI:** 10.12688/f1000research.73240.1

**Published:** 2021-10-25

**Authors:** Kyle Ong, Kok-Why Ng, Su-Cheng Haw

**Affiliations:** 1 Faculty of Computing and Informatics, Multimedia University, Persiaran Multimedia, Cyberjaya, Selangor, 63100, Malaysia

**Keywords:** Recommender System, Matrix Factorization, Collaborative Filtering, Deep Neural Networks, Neural Collaborative Filtering.

## Abstract

In recent years, Recommender System (RS) research work has covered a wide variety of Artificial Intelligence techniques, ranging from traditional Matrix Factorization (MF) to complex Deep Neural Networks (DNN). Traditional Collaborative Filtering (CF) recommendation methods such as MF, have limited learning capabilities as it only considers the linear combination between user and item vectors. For learning non-linear relationships, methods like Neural Collaborative Filtering (NCF) incorporate DNN into CF methods. Though, CF methods still suffer from cold start and data sparsity. This paper proposes an improved hybrid-based RS, namely Neural Matrix Factorization++ (NeuMF++), for effectively learning user and item features to improve recommendation accuracy and alleviate cold start and data sparsity. NeuMF++ is proposed by incorporating effective latent representation into NeuMF via Stacked Denoising Autoencoders (SDAE). NeuMF++ can also be seen as the fusion of GMF++ and MLP++. NeuMF is an NCF framework which associates with GMF (Generalized Matrix Factorization) and MLP (Multilayer Perceptrons). NeuMF achieves state-of-the-art results due to the integration of GMF linearity and MLP non-linearity. Concurrently, incorporating latent representations has shown tremendous improvement in GMF and MLP, which result in GMF++ and MLP++. Latent representation obtained through the SDAEs’ latent space allows NeuMF++ to effectively learn user and item features, significantly enhancing its learning capability. However, sharing feature extractions among GMF++ and MLP++ in NeuMF++ might hinder its performance. Hence, allowing GMF++ and MLP++ to learn separate features provides more flexibility and greatly improves its performance. Experiments performed on a real-world dataset have demonstrated that NeuMF++ achieves an outstanding result of a test root-mean-square error of 0.8681. In future work, we can extend NeuMF++ by introducing other auxiliary information like text or images. Different neural network building blocks can also be integrated into NeuMF++ to form a more robust recommendation model.

## Introduction

Collaborative Filtering (CF) based Recommender System (RS) typically suggests items based on user-item interactions. Users’ interests are predicted based on analyzing other users’ tastes and preferences in the system. Matrix Factorization (MF), ^1^ popularized by the Netflix price, ^2^ has emerged as a powerful CF recommendation tool. However, its simple interaction function, which is the inner product, has hindered its performance. Not to mention that CF methods also suffer from cold start and data sparsity.

Much effort has been devoted to improving MF’s accuracy throughout the years, but one approach that has caught much attention is deep learning (DL). DL has drastically improved MF’s accuracy by exploiting deep neural networks (DNN). Eventually, many researchers have also suggested incorporating side information into CF methods. This subsequently forms a hybrid-based (HB) method that solves CF’s cold start and data sparsity. ^3^

In this paper, we proposed a novel hybrid-based RS named Neural Matrix Factorization ++ (NeuMF++). NeuMF++ is an improved version of NeuMF that incorporates an effective latent representation of side information via Stacked Denoising Autoencoders (SDAEs). In the original work, NeuMF has achieved outstanding results. It is surprising to see that not much prior work has been done to enhance NeuMF. In NeuMF++, SDAEs extract high-level representations from side information and later incorporate them as latent feature vectors. Incorporating user-item features in the learning process enhances its learning capabilities and improves its recommendation performance. Experiments on a real-world dataset have demonstrated the effectiveness of side information in NeuMF++, yielding state-of-the-art results.

The rest of the paper is organized as follows. Section 2 discusses the related work. Section 3 introduces our proposed framework, NeuMF++, in detail. Section 4 discusses the result. Finally, section 5 summarizes the paper and briefly introduces our future work.

## Related work

There are different DL models ranging from standard Multilayer Perceptrons (MLP) to Convolutional Neural Network (CNN). DL models like MLP are utilized to add the non-linear transformation to existing linear techniques and interpret them as neural extensions. ^4^^,^^5^ NCF frameworks, ^2^ which include Generalized MF (GMF), MLP and NeuMF, make use of DNN into traditional MF to further enhance its recommendation performance and quality. The differences between the three models are their interaction functions. GMF uses a linear kernel by taking user and item latent vectors and multiplying them element by element (element-wise product). In contrast, MLP uses a non-linear kernel by concatenating user and item latent vectors and then fully connects to an MLP. Lastly, NeuMF integrates the linearity of GMF and non-linearity MLP by combining both of their outputs with a single-layer MLP.

Another popular DL model is the Autoencoder (AE). AE is a powerful tool for dimensionality reduction and can be considered a strict generalization of Principal Component Analysis. It aims to reconstruct the input data as output. Many popular MF techniques can be thought of as a form of dimensionality reduction. ^3^ Therefore, AE can be adapted for this task as well, such as AutoRec. ^6^ Subsequently, ^7^ further enhances AutoRec by training it much deeper, which aids the network to generalize better ^8^ proposed Collaborative Denoising Autoencoder, which utilized a Denoising Autoencoder (DAE) to perform CF tasks. Noises are added intentionally to the rating input and reconstructing the original rating input as the output. This allows the network to be more noise-resistant and helps it to learn more stable features.

Most studies only focus on ratings, but ratings alone are unable to reveal user-item relation fully. Additionally, most CF methods also suffer from cold start and data sparsity. Hence, several researchers suggested incorporating side information into the model, forming an HB method ^3^^,^^8^ proposed a new HB method known as CF Network (CFN). Instead of only adding the side information into the first layer, the author injected that information into every layer except the output layer of the network.

However, most AE-based CFs utilize side information as regularization in their models. However, due to the sparse nature of the rating matrix together with side information, the learned latent vectors might not be very effective. Therefore, ^9^ introduced Collaborative Deep Learning (CDL), in which a DAE learns item features and is then utilized as an item latent vector for MF. Subsequently, ^10^ proposed a marginalized DAE for CF (mDA-CF), an extension of CDL by adding user latent vectors learned by another AE. The key of mDA-CF is to extract user and item features from mDAs and combine them in a joint framework.

Even though both CFL and mDA-CF utilize DNN to improve recommendation performance, their CF’s core is still a linear MF. Therefore, ^3^ proposed two models 一 GMF++ and MLP++. GMF++/MLP++ enhances the GMF/MLP of the NCF frameworks by incorporating user and item latent vectors extracted from SDAEs into neural collaborative filtering.

## Methods

The real-world dataset was obtained from the GroupLens Research Project. The GroupLens Research Project is a research group in the Department of Computer Science and Engineering at the University of Minnesota. The Movielens-1M dataset from the GroupLens Research is available at: https://grouplens.org/datasets/movielens/1m/.

Ethical Approval Number: EA1572021

Ethical Approval Body: Research Ethic Committee 2021, Multimedia University

First, we will present NeuMF++ as a general framework. Then, we will describe feature extraction and neural collaborative filtering in detail. Lastly, we will explain the learning and optimization of NeuMF++. Table 1 shows the frequent notations.

**Table 1.** Frequent notation.

Notation	Description
m	Number of users.
n	Number of items.
d	Embedding dimension.
p	User feature dimension.
q	Item feature dimension.
$X\in R^{m\times q}$	User side information.
$V\in R^{n\times q}$	Item side information.
$P\in R^{m\times d}$	User embedding.
$Q\in R^{n\times d}$	Item embedding.
r	Rating.
$\sigma_{l}$	Non-linear function at layer-l.
$W_{l}$	Weight matrix at layer-l.
$b_{l}$	Bias matrix at layer-1.
$p_{u}$	User latent vector.
$q_{i}$	Item latent vector.

### NeuMF++: A general framework

**Figure 1.**
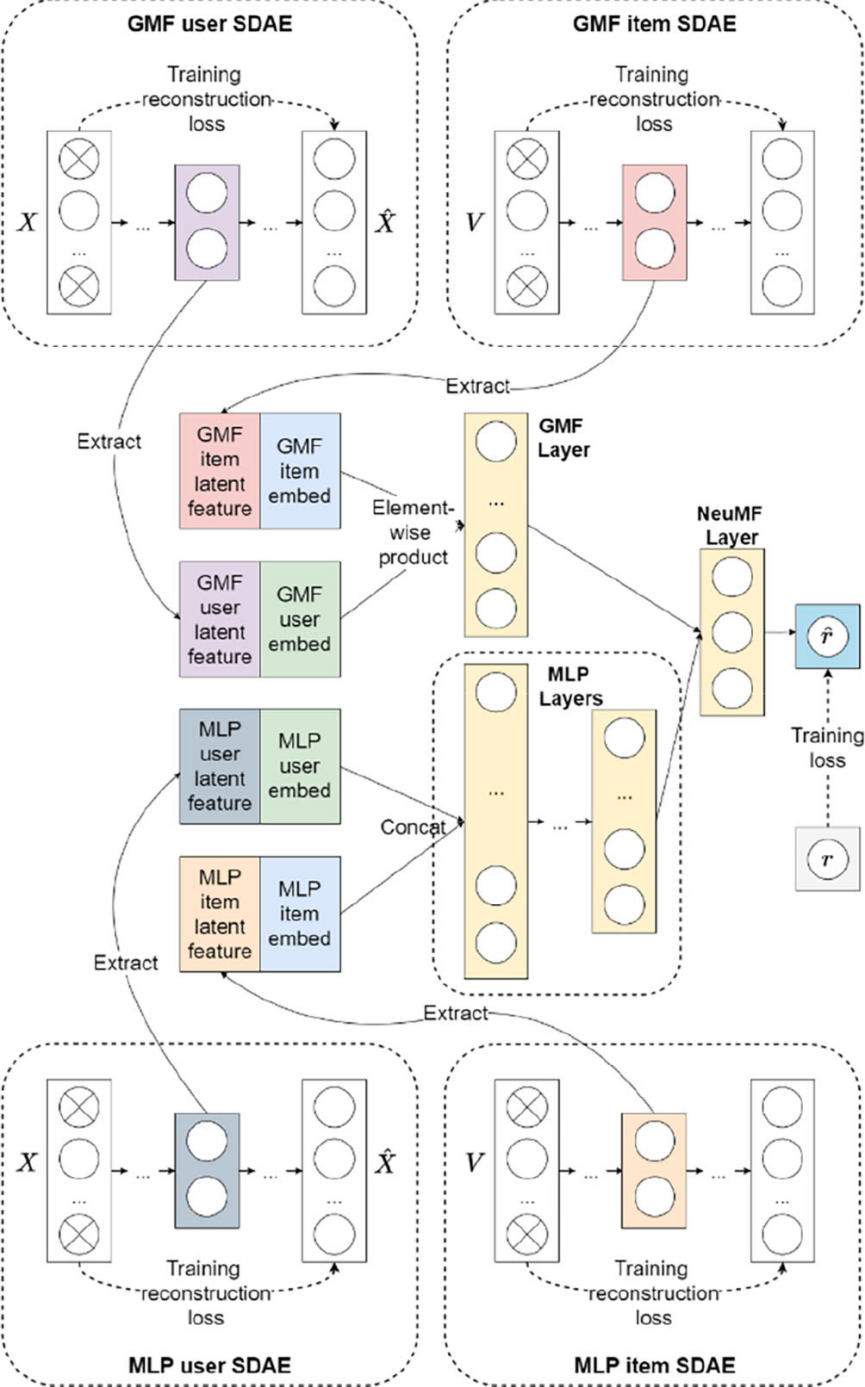
NeuMF++ architecture

In this section, the proposed NeuMF++ is introduced in general. As illustrated in Figure 1, NeuMF++ is a hybrid model that bridges multiple SDAEs to a NeuMF. NeuMF++ contains two major components: feature extraction and neural collaborative filtering.

In feature extraction, each user and item features are assigned with 2 SDAEs for feature extraction. As discussed earlier, recommendation performance and accuracy can be improved by incorporating side information. NeuMF++ utilizes SDAEs to learn user-item features by minimizing the errors of the reconstructed and the original input features. Then, compressed high-level features can be extracted from the bottleneck layer, located in the middle-most layer. In neural collaborative filtering, NeuMF has been chosen as our framework due to its outstanding performance. As mentioned earlier, NeuMF combines the output of GMF and MLP interaction functions. Similarly, NeuMF++ combines the output of GMF++ and MLP++ interaction functions. First, user and item latent vectors can be formed by concatenating the user and item embeddings of GMF and MLP, with the learned user and item latent feature vectors extracted from the SDAEs. Then, the user and item latent vector will be fed to the respective GMF++ and MLP++ interaction function. Finally, the outputs obtained from GMF++ and MLP++ are concatenated and fed into a single-layer MLP 一 NeuMF layer to generate ratings.

### NeuMF++: Feature extraction

SDAE can be formed by stacking multiple DAEs on top of one another. Side information (features) is usually composed of the subject attributes like users’ age and occupation or item’s shape and size. In NeuMF++, SDAEs take user features X and item features V as input, encode them in a low-dimensional latent space, and then reconstruct $\hat{X}$ and $\hat{V}$ in the output space. At the same time, noises are added intentionally between layers during training.

For example, given a set of features $X\in R^{m\times p}$ the SDAE minimize the reconstruction error,

$$l_{u}={\left\|X-\hat{X}\right\|}_{F}^{2}+\lambda_{\omega }{\left\|\omega \right\|}_{F}^{2}$$ (1)

where ω denotes as the model parameters, $\lambda_{\omega }$ as the regularization term, and $\hat{X}$ as the reconstruction of $X\in R^{m\times p}$ , where

$$\hat{X}=\sigma_{L}\left(\nabla \left(\ldots \nabla \left(\sigma_{1}\left(\bar{X}W_{1}^{X}+b_{1}^{X}\right)\right)\ldots \right)\right)W_{L}^{X}+b_{L}^{X}$$ (2)

where ∇ denotes the noise function. During inference, the values of the bottleneck layer can be extracted as in Eq. (3).

$$X_{\left(L^{X}/2\right)}=\sigma_{\left(L/2\right)}\left(\ldots \sigma_{1}\left({\mathrm{XW}}_{1}^{X}+b_{1}^{X}\right)\ldots \right)W_{\left(L/2\right)}^{X}+b_{\left(L/2\right)}^{X}$$ (3)

### NeuMF++: Neural collaborative filtering

NeuMF++ can be seen as the combination of GMF++ and MLP++. The ++ acronym denotes that side information is appended to the model. At first, one-hot encoding is performed on user and item ID to obtain the user and item embeddings. Then, user and item latent feature vectors are extracted and concatenated with their respective embedding to form user and item latent vectors $p_{u}$ and $q_{i}$ , formulated as such

$$p_{u}=\left[\begin{array}{c} P^{u}\\ X_{\left(L^{X}/2\right)}^{u} \end{array}\right]$$ (4)

$$q_{i}=\left[\begin{array}{c} Q^{i}\\ V_{\left(L^{V}/2\right)}^{i} \end{array}\right]$$ (5)

As discussed earlier, GMF++ and MLP++ use different computations and layers in their interaction function. GMF++ performs an element-wise product between $p_{u}$ and $q_{i}$ as shown in Eq. (6). In contrast, MLP++ utilizes a standard MLP by adding several hidden layers on the concatenated latent vectors, as shown in Eq. (7).

$$\phi^{\left(\mathrm{GMF}++\right)}=p_{u}⊙q_{i}$$ (6)

$$\phi^{\left(\mathrm{MLP}++\right)}=\sigma_{L}\left(\ldots \sigma_{1}\left(\left[\begin{array}{c} p_{u}\\ q_{i} \end{array}\right]W_{1}+b_{1}\right)\ldots \right)W_{L}+b_{L}$$ (7)

Finally, the NeuMF layer, a single-layer MLP, is introduced to combine both GMF++ and MLP++ interaction output. Specifically, NeuMF++ integrates GMF++ and MLP++ with a single-layer MLP can be formulated in Eq. (8).

$$\hat{r}=\sigma \left(\left[\begin{array}{c} p_{u}⊙q_{i}\\ \sigma_{1}\left(\left[\begin{array}{c} p_{u}\\ q_{i} \end{array}\right]W_{1}+b_{1}\right) \end{array}\right]W+b\right)$$ (8)

From Eq. (8), we can see that GMF++ and MLP++ shared the same $p_{u}$ and $q_{i}$ which extracted from the same user and item SDAEs. This might limit the performance and learning capabilities of NeuMF++. For example, the hyperparameters and latent vector size between GMF++ and MLP++ might vary. Hence, we allow GMF++ and MLP++ to perform user-item feature extraction separately. This provides more flexibility to the NeuMF++. Hence, the final NeuMF++ algorithm can be written as,

$$\phi^{\left(\mathrm{GMF}++\right)}=p_{u}^{\left(\mathrm{GMF}++\right)}⊙q_{i}^{\left(\mathrm{GMF}++\right)}$$ (9)

$$\phi^{\left(\mathrm{MLP}++\right)}=\sigma_{L}\left(\ldots \sigma_{1}\left(\left[\begin{array}{c} p_{u}^{\left(\mathrm{MLP}++\right)}\\ q_{i}^{\left(\mathrm{MLP}++\right)} \end{array}\right]W_{1}+b_{1}\right)\ldots \right)W_{L}+b_{L}$$ (10)

$$\hat{r}=\sigma \left(\left[\begin{array}{c} \phi^{\left(\mathrm{GMF}++\right)}\\ \phi^{\left(\mathrm{MLP}++\right)} \end{array}\right]W+b\right)$$ (11)

### NeuMF++: Learning and optimization

NeuMF++ objective function consists of user-item feature reconstruction error in feature extraction and prediction error in neural collaborative filtering. The loss function of user and item SDAE can be seen in Eq. (1). Since NeuMF++ is a rating prediction model, its output $\hat{r_{\mathrm{ui}}}$ range between $\left[0,N\right]$ Where N is the maximum rating number. Hence, the loss function can be defined in Eq. (12),

$$l_{r}={\left\|r_{\mathrm{ui}}-\hat{r_{\mathrm{ui}}}\right\|}_{F}^{2}+\lambda_{\theta }{\left\|\theta \right\|}_{F}^{2}$$ (12)

where θ denotes as the parameters of the models, $\lambda_{\theta }$ as the regularization term.

Therefore, the general loss function for optimizing NeuMF++ is formulated in Eq. (13).

$$l=l_{r}+\alpha l_{u}^{\left(\mathrm{GMF}++\right)}+\beta l_{i}^{\left(\mathrm{GMF}++\right)}+\gamma l_{u}^{\left(\mathrm{MLP}++\right)}+\delta l_{i}^{\left(\mathrm{MLP}++\right)}$$ (13)

where α,β,γ,δ are trade-off parameter for each reconstruction loss.

## Results

### Experimental settings

This paper uses the public MovieLens 1-M dataset. ^11^ The dataset contains approximate 1 million ratings from 6040 unique users across 3706 unique movies, with 95.8% sparseness. Concurrently, we also use the side information provided by the dataset. The user side information consists of age, occupation and gender attributes, while the item consists of 18 different movie genres. All features are preprocessed and encoded as one-hot numeric arrays.

The evaluation index used in this paper is the root mean square error, RMSE, as shown in Eq. (14). RMSE is directly related to our loss function. The smaller the RMSE, the better the recommendation accuracy.

$$\text{RMSE}=\sqrt{\sum_{u=1}^{m}\sum_{i=1}\left(\frac{\left(r_{\mathrm{ui}}-\hat{r_{\mathrm{ui}}}\right)}{N}\right)}$$ (14)

We compared our proposed NeuMF++ with related baseline models which include MF, GMF, MLP, NeuMF, GMF++ and MLP++. ^1^^-^^3^

All the experiments were implemented using Pytorch, a deep learning framework built on top of the Python programming language. We utilized the Adam optimization method to optimize our model by setting the batch size of 1024, regularization term of 0.001 and learning rate of 0.001. Concurrently, we split the dataset into 70:30 ratios, where 70% of the dataset is used for training, while another 30% is used for testing. The hyperparameters used on the related baseline models are based on their respective publications. ^2^^,^^3^

As mentioned previously, we used different hyperparameters on GMF++ and MLP++ for user-item feature extraction. We used 8 neurons on 1 hidden layer in GMF++ user-item SDAEs, and 16:8:16 neurons on 3 hidden layers in MLP++ user-item SDAEs. Hence, the latent vector dimensions for all SDAEs are 8. Each SDAE layer is also inputted with some Gaussian noises. In neural collaborative filtering, the embedding vector dimension, d chosen is 8. We used ReLU as GMF++ activation function, while SeLU as MLP++ activation function. Concurrently, MLP++ composed of [32,16,8] neurons in its interaction MLP layers. Finally, we set all the trade-off parameters α,β,γ,δ to 0.000001.

### Experimental result and analysis

In Table 2, we can see that NeuMF++ has proved to outperform all the other baseline models with 0.7964 in train RMSE and 0.8681 in test RMSE. NeuMF++ has achieved a 1.37% improvement than its predecessor NeuMF and 2% improvement than traditional MF. As a result, NeuMF++ has demonstrated the effectiveness of employing DNN and side information for rating prediction.

**Table 2.** RMSE of different compared models on 1M Movielens data with 70-30 train-test split.

Method	Training RMSE	Testing RMSE
MF	0.8010	0.8958
GMF	0.7835	0.8928
GMF++	0.7738	0.8894
MLP	0.8696	0.8879
MLP++	0.8686	0.8864
NeuMF	0.8152	0.8725
**NeuMF++ (Ours)**	0.7964	0.8681

**Figure 2.**
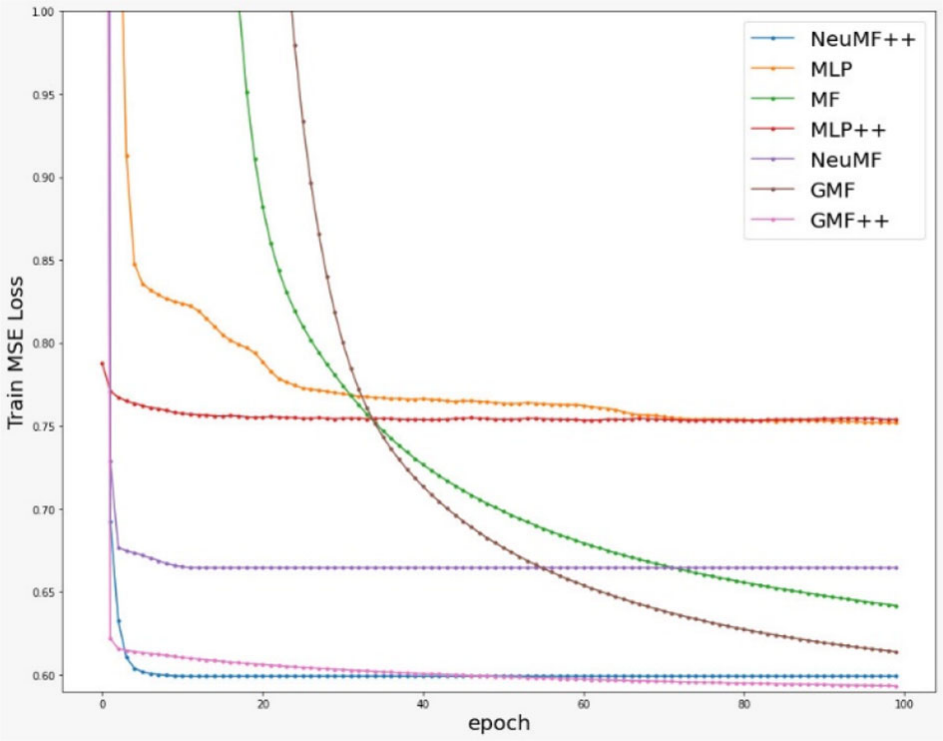
Training loss of compared models over 100 iterations/epochs.

**Figure 3.**
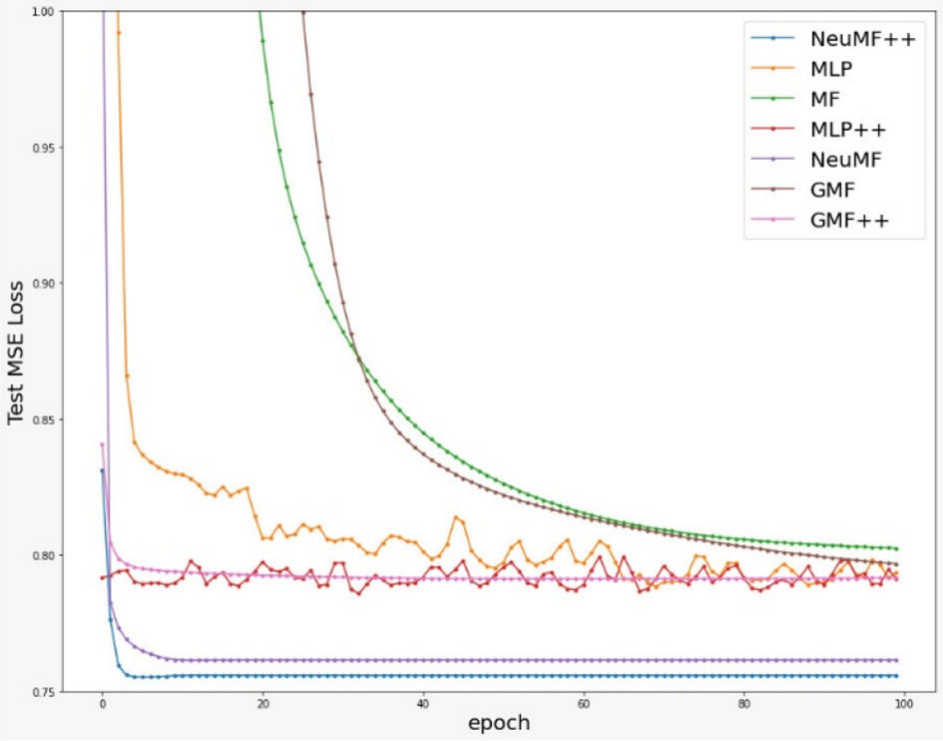
Testing loss of compared models over 100 iterations/epochs.

Figures 2 and 3 show that most models converged very fast, except for MF and GMF. This shows that models with DNN learn much faster than the models without DNN in this dataset. Also, MLP++ does not converge as much as MLP. Therefore, side information does not provide much effect on MLP.

To demonstrate the effectiveness of separate feature extraction and pre-trained weights for NeuMF++, we compared the performance on three versions of NeuMF++ as seen in Table 3. As expected, NeuMF++, with pre-trained weights and feature extraction separated among the GMF++ and MLP++ layers, achieve the best performance.

**Table 3.** RMSE of different NeuMF variations on 1M Movielens data with 70-30 train-test split.

Method	Training RMSE	Testing RMSE
NeuMF	0.8152	0.8725
NeuMF++	0.8686	0.8865
NeuMF++ (seperate)	0.9007	0.9108
**NeuMF++ (seperate + pre-train)**	0.7964	0.8681

**Figure 4.**
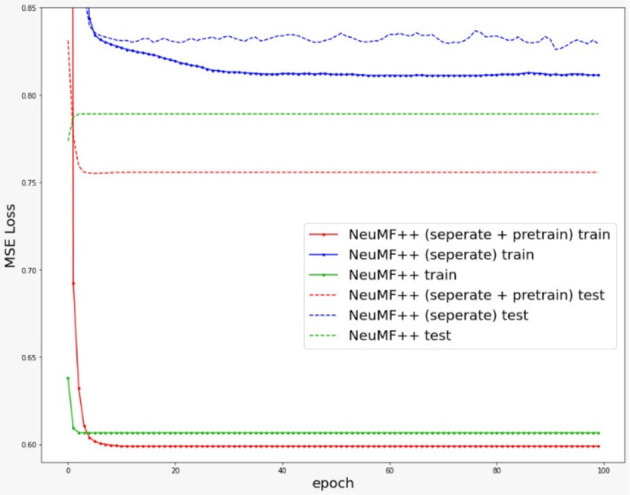
Training and testing loss of different NeuMF variations over 100 iterations/epochs.

Concurrently, we also observed that NeuMF++ with feature extraction shared among the GMF and MLP layers, over-fitted in the early iterations, as shown in Figure 4.

At first, we found out that NeuMF++ did not perform as well as NeuMF. Hence, inspired by the concept of a pre-training method from, ^2^ we loaded and froze pre-trained GMF++ and MLP++ weights into NeuMF++. As a result, we noticed a 8.11% improvement, as shown in Table 3. This pre-training method updates weights within the NeuMF layer but not within the GMF++ and MLP++ layers. As a result, NeuMF++ with pre-trained weights performed much better as compared to NeuMF++ without pre-trained weights. This justified that the usefulness of the pre-training method for initializing NeuMF++.

## Conclusion

In this paper, we proposed an HB recommendation model, namely NeuMF++, which is an enhanced version of NeuMF that incorporates effective latent representations of side information. Throughout the experiment, we found that incorporating side information to neural collaborative filtering can improve the recommendation performance and eliminate CF cold start and data sparsity.

NeuMF++ is also not limited to categorical or numerical type information, and can be extended with other information types such as text or even images. For example, pre-trained word embedding models such as word2vec, ELMO or BERT, can transform textual information into input bags of words. Besides, CNN can also learn features from images and aid feature extraction or neural collaborative filtering.

DL’s flexibility also allows different neural network building blocks to be integrated. This concept can also be applied to NeuMF++ to form a more robust recommendation model and further improve its recommendation precision.

## Author contributions

Ong, Ng and Haw conceived the presented idea. Ong carried out the experiment and wrote the manuscript. Ng and Haw supervised the project and provided critical feedback.

## Data availability

None.
