# Supplementary material for: Neural matrix factorization++ based recommendation system
Source: F1000Res. 2021 Oct 25;10:1079. [Version 1] doi: 10.12688/f1000research.73240.1 (PMC10973760; doi:10.12688/f1000research.73240.1)
Supplement: Peer review history

# Reviewer report: Reviewer response for version 1

**DOI:** 10.5256/f1000research.76881.r211206

**Published:** 2024-03-27

**Authors:** Zhigang Liu

**Affiliations:** 1 Chongqing Institute of Green and Intelligent Technology, Chinese Academy of Sciences, Chongqing University of Posts and Telecommunications,, Chongqing, China

This paper proposes a hybrid recommender system based on neural matrix factorization and stacked denoising autoencoders. The authors claim that their model, called NeuMF++, can effectively learn user and item features from side information and improve the accuracy of rating prediction. The proposed method was evaluated on a real-world dataset and compare it with several baseline methods, showing that NeuMF++ achieves the best performance in terms of root mean square error. In general, this study is interesting and has some significance to recommender system community. However, considering its current form, I have some major concerns for the authors to address:

 (1) The paper lacks a clear motivation and contribution statement. The authors should explain why they choose to combine neural matrix factorization and stacked denoising autoencoders, and what are the main advantages and challenges of their approach. The authors should also highlight how their work differs from the existing literature and what are the novel aspects of their model.

 (2) The paper should provide a more comprehensive literature review that covers more related work on MF-based recommendation systems, and latent factor analysis, e.g., Professor Xin Luo’s work. The paper should also highlight the novelty and significance of the proposed method in relation to existing work.

 (3) The paper does not provide enough details and justification for the design choices and hyperparameters of the model. For example, the authors should explain why they use different activation functions, noise functions, and latent vector dimensions for GMF++ and MLP++. The authors should also report how they tune the trade-off parameters α, β, γ, δ and the regularization terms *λ*ω and *λ*θ, and what are the effects of these parameters on the model performance.

 (4) The paper does not conduct a comprehensive evaluation of the model. The authors only use one very small dataset and one metric to assess their model, which limits the generalizability and robustness of their results. The authors should also perform ablation studies to analyze the contribution of each component of their model.

 (5) The paper does not discuss the limitations and future directions of their work. The authors should acknowledge the potential drawbacks and challenges of their model, such as scalability, interpretability, cold start, etc.

Is the work clearly and accurately presented and does it cite the current literature?

Yes

If applicable, is the statistical analysis and its interpretation appropriate?

No

Are all the source data underlying the results available to ensure full reproducibility?

Partly

Is the study design appropriate and is the work technically sound?

Partly

Are the conclusions drawn adequately supported by the results?

Yes

Are sufficient details of methods and analysis provided to allow replication by others?

Partly

Reviewer Expertise:

Latent factor analysis, community detection

I confirm that I have read this submission and believe that I have an appropriate level of expertise to confirm that it is of an acceptable scientific standard, however I have significant reservations, as outlined above.

# Reviewer report: Reviewer response for version 1

**DOI:** 10.5256/f1000research.76881.r211221

**Published:** 2023-10-16

**Authors:** Khanh Luong

**Affiliations:** 1 Queensland University of Technology, Brisbane, Australia

This paper introduces a novel Hybrid recommendation model that leverages two distinct models: GMF (Generalized Matrix Factorization) and SDAE (Stacked Denoising Autoencoder). This hybrid model is composed of two fundamental components: feature extraction and collaborative filtering, where feature extraction step was done on both user and item data (using both GMF and SDAE).

 The underlying concept behind this hybrid model is straightforward yet promising. The paper's structure and writing are well-organized and easily comprehensible.

 Experiment has been done and reported the RMSE on both training and testing data, however, it is crucial to measure the accuracy of the proposed hybrid model against benchmark models. Therefore, it is suggested that authors provide accuracy comparisons in their paper in order to strengthen its rigour and provide a more comprehensive picture of the hybrid proposed model.

Is the work clearly and accurately presented and does it cite the current literature?

Partly

If applicable, is the statistical analysis and its interpretation appropriate?

Not applicable

Are all the source data underlying the results available to ensure full reproducibility?

Yes

Is the study design appropriate and is the work technically sound?

Yes

Are the conclusions drawn adequately supported by the results?

Partly

Are sufficient details of methods and analysis provided to allow replication by others?

Yes

Reviewer Expertise:

unsupervised learning, dimensionality reduction, multi-view/multi-modal learning

I confirm that I have read this submission and believe that I have an appropriate level of expertise to confirm that it is of an acceptable scientific standard, however I have significant reservations, as outlined above.

# Reviewer report: Reviewer response for version 1

**DOI:** 10.5256/f1000research.76881.r101096

**Published:** 2022-02-15

**Authors:** Dino Caesaron

**Affiliations:** 1 Telkom University, Bandung, Indonesia

Overall, the work is fine, and is suitable for indexing. The experimental design has been meticulously planned. The proposed methodology is an improvement to the existing work. The experiment tested on 1 million movielens data. Hence, the proposed method is considered robust and stable. The only weakness is that perhaps it would be useful to test on data obtained from different domains.

 Please include the benefit of this research's finding in the abstract as well as in the introduction sections, if possible.

 I recommend indexing this article with minor revisions or as is.

Is the work clearly and accurately presented and does it cite the current literature?

Yes

If applicable, is the statistical analysis and its interpretation appropriate?

Yes

Are all the source data underlying the results available to ensure full reproducibility?

Yes

Is the study design appropriate and is the work technically sound?

Yes

Are the conclusions drawn adequately supported by the results?

Yes

Are sufficient details of methods and analysis provided to allow replication by others?

Yes

Reviewer Expertise:

virtual/augmented reality, human factors engineering

I confirm that I have read this submission and believe that I have an appropriate level of expertise to confirm that it is of an acceptable scientific standard.
